# Development and optimization of sitagliptin and dapagliflozin loaded oral self-nanoemulsifying formulation against type 2 diabetes mellitus

**DOI:** 10.1080/10717544.2020.1859001

**Published:** 2020-12-21

**Authors:** Mohsin Kazi, Abdulmohsen Alqahtani, Ajaz Ahmad, Omar M. Noman, Mohammed S. Aldughaim, Ali S. Alqahtani, Fars K. Alanazi

**Affiliations:** a Department of Pharmaceutics, College of Pharmacy, King Saud University, Riyadh, Saudi Arabia; b Department of Clinical Pharmacy, College of Pharmacy, King Saud University, Riyadh, Saudi Arabia; c Medicinal Aromatic, and Poisonous Plants Research Center, College of Pharmacy, King Saud University, Riyadh, Saudi Arabia; d Research Center, King Fahad Medical City, Riyadh, Saudi Arabia; e Department of Pharmacognosy, College of Pharmacy, King Saud University, Riyadh, Saudi Arabia

**Keywords:** Self-nanoemulsifying drug delivery systems (SNEDDS), antioxidant activity, sitagliptin and dapagliflozin, combined dosage form, oral bioavailability enhancement, glucose inhibition

## Abstract

Control of hyperglycemia and prevention of glucose reabsorption (glucotoxicity) are important objectives in the management of type 2 diabetes. This study deals with an oral combined dosage form design for two anti-diabetic drugs, sitagliptin and dapagliflozin using self-nanoemulsifying drug delivery systems (SNEDDS). The SNEDDS were developed using naturally obtained bioactive medium-chain/long-chain triglycerides oil, mixed glycerides and nonionic surfactants, and droplet size was measured followed by the test for antioxidant activities. Equilibrium solubility and dynamic dispersion experiments were conducted to achieve the maximum drug loading. The *in vitro* digestion, *in vivo* bioavailability, and anti-diabetic effects were studied to compare the representative SNEDDS with marketed product Dapazin^®^. The representative SNEDDS containing black seed oil showed excellent self-emulsification performance with transparent appearance. Characterization of the SNEDDS showed nanodroplets of around 50–66.57 nm in size (confirmed by TEM analysis), in addition to the high drug loading capacity without causing any precipitation in the gastro-intestinal tract. The SNEDDS provided higher antioxidant activity compared to the pure drugs. The *in vivo* pharmacokinetic parameters of SNEDDS showed significant increase in *C*_max_ (1.99 ± 0.21 µg mL^−1^), AUC (17.94 ± 1.25 µg mL^−1^), and oral absorption (2-fold) of dapagliflozin compared to the commercial product in the rat model. The anti-diabetic studies showed the significant inhibition of glucose level in treated diabetic mice by SNEDDS combined dose compared to the single drug therapy. The combined dose of sitagliptin-dapagliflozin using SNEDDS could be a potential oral pharmaceutical product for the improved treatment of type 2 diabetes mellitus.

## Graphical Abstract

**Figure:**
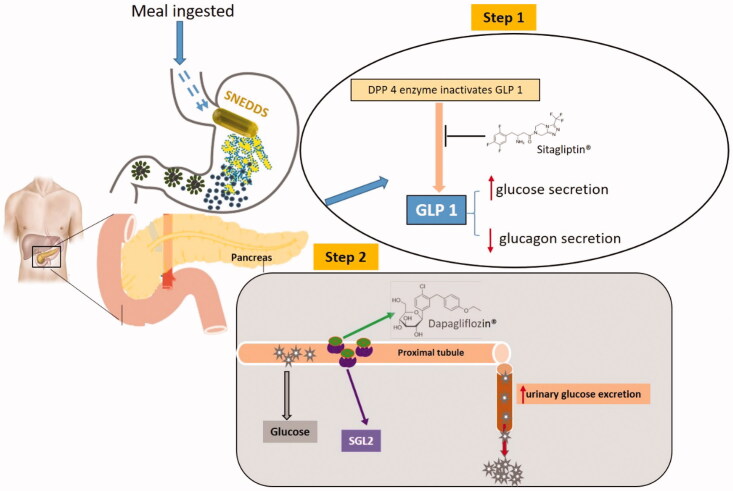

Schematic diagram shows the proposed mechanism of action of anti-diabetic compounds sitagliptin and dapagliflozin from the SNEDDS combined dosage form.

## 1. Introduction

Type 2 diabetes is a progressive disease that is characterized with increasing blood glucose levels over time. The disease is a major cause of blindness, heart attacks, stroke, kidney failure, and lower limb amputation in patients (Al Dawish et al., 2016). Most of the type 2 diabetic patients require multiple therapies in order to control their blood sugar levels effectively and maintain glycemia at or below the A1c target of less than 7% (Al-Faris et al., 2006). Although there have been several studies performed to evaluate the effectiveness of combined oral therapy to improve glycemic control, there is no large-scale definitive study on which of these are the best successive combinations to use. Therefore, emphasis should be given to the new combination therapies of anti-diabetic drugs, which must keep the A1c from rising much above the target range (Charpentier, 2002).

Occurrence of type 2 diabetes is increasing globally, currently affecting almost 450 million people worldwide (8.8% of the world population), according to the International Diabetes Federation (Mathers & Loncar, 2006; Lopez & Mathers, 2006) and causing a great economic burden on health care systems due to its adverse effect on people’s health. Saudi Arabia is reported to be one of the seven leading countries in the world, with an estimated 2.3 million people being diagnosed with the disease (Al Dawish et al., 2016).

The use of combinational therapy often results in reduction in blood glucose levels compared to those of any single drugs available or oral monotherapy (Yki-Jarvinen, 2002). A wide range of combinations such as thiazolidinedione, or acarbose; sulfonylurea plus metformin, metformin plus a thiazolidinedione or acarbose have been used effectively to achieve glycemic control in patients for whom oral monotherapy had failed and therefore demand for combination therapy (Charpentier, 2002; Charbonnel et al., 2006; Aquilante et al., 2007). In addition, increasing line of evidence suggests that patients who are not taking insulin, combination therapy involving oral anti-diabetic agents characterized with different mechanisms of actions may be effective in controlling blood glucose levels (Nichols et al., 2007). Low-dose combinational therapy is believed to have the potential to reduce the occurrence of side effects associated with higher doses of single agents and may also achieve superior glycemic control (Vahatalo et al., 2007).

**Figure 1.**
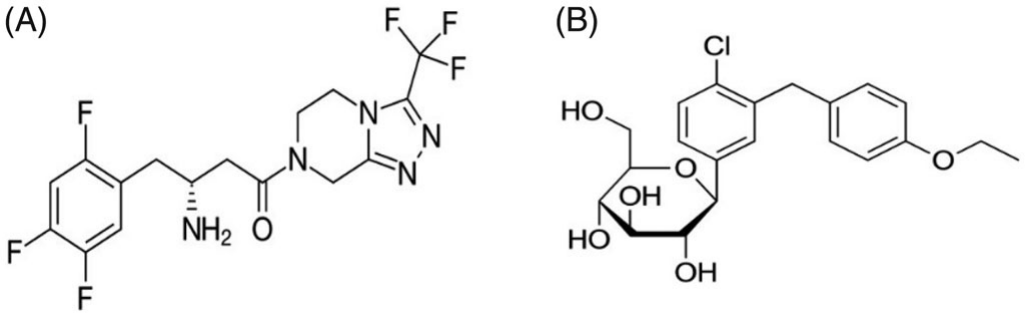
Chemical structure of (A) sitagliptin (MW: 407.32 g/mol), (B) dapagliflozin (MW: 408.875 g/mol).

Sitagliptin (SN), an orally active, potent dipeptidyl-peptidase inhibitor [DPP-4 inhibitor (Figure 1(A)) has recently been approved (listed in top 20 drugs in 2017) for the therapy of type 2 diabetes (Zerilli & Pyon, 2007). Like other DPP-4 inhibitors, its mechanism of action is by increasing glucagon-like peptide-1 incretin hormones and gastric inhibitory polypeptide (Miller et al., 2009). SN is effective in lowering HbA1c, and glucose (during fasting as well as postprandial) in monotherapy and in combination with other oral anti-diabetic agents (Plosker, 2014; Dey, 2017). It stimulates insulin secretion when hyperglycemia is present and inhibits glucagon secretion (Scheen & Van Gaal, 2013).

Dapagliflozin (DN; Figure 1(B)) is a sodium glucose co-transporters (SGLTs inhibitor) which acts in transporting glucose, vitamins, amino acids, osmolytes, and ions across tissue membranes and SGLT1 and SGLT2 mediate glucose reabsorption (Plosker, 2012; Del Prato et al., 2015). While SGLT1 is the main glucose transporter in the small intestine, SGLT2 is predominantly expressed on epithelial cells surface on the lining of the S1 segment of the proximal convoluted tubule (Fioretto & Avogaro, 2017). Studies have shown DN to be the most selective for SGLT2 (1200-fold over SGLT1), which could be the better drug for the treatment of type 2 diabetes (Santos et al., 2017; Wilding et al., 2017).

SGLT2 inhibitors such as DN and dipeptidyl peptidase-4 (DPP-4) inhibitors like SN is potentially beneficial in achieving glycemic control without the risk of hypoglycemia or weight gain treatment of type 2 diabetes (Santos et al., 2017). A recent study suggests that in patients with type 2 diabetes, combination therapy (Ji et al., 2016) provided additional clinical benefit and is well tolerated. Reductions in HbA1c and body weight with DN were observed in patients receiving SN either alone or in combination with metformin (Gallwitz, 2007; Shimoda et al., 2013).

The physicochemical characteristics of SN and DN suggest that they are suitable for self-nanoemulsifying drug delivery system (SNEDDS) as such system is reported to improve drug solubility, permeability, and bioavailability after oral administration (Kazi et al., 2017; Pandey et al., 2020). Liquid SNEDDS can be easily filled into soft/hard gelatin capsules and converted into solid dosage form in a single step process via spray drying, adsorption techniques. Both liquid and solid SNEDDS have been shown to enhance the drug release significantly and maintain the drug in solubilized form during the GI transit time (Khursheed et al., 2020). In a recent study, the biochemical, hematological and histopathological investigations in diabetic rats showed the potential application of SNEDDS when loaded with Polypeptide K as anti-diabetic drug (Garg et al., 2017). SNEDDS in the current studies were developed using black seed oil (BSO) which contained therapeutically active compound thymoquinone. (a phytochemical shows anti-inflammatory and antioxidant effects). Thymoquinone is reported to have anti-inflammatory and antioxidant effects and thus could show the synergistic effects along with the SN-DN combined dose.

This technique of SD-DN SNEDDS development can be highly efficient as it could improve solubility and produce stable nanodroplets without causing any precipitation upon aqueous dilution with the GIT fluids before absorption. Therefore, the aim of the current research was to prepare an effective combined oral dosage form of SN and DN (encapsulated in liquid SNEDDS) with improved anti-diabetic effects to meet the current need as improved treatment of type 2 diabetes.

## 2. Materials and methods

### 2.1. Materials

The model drugs, SN (purity 99.9%) and DN, reference standard (purity >99.8%) were obtained as a gift from Renata (former Pfizer), and Concord Pharmaceuticals Ltd (Dhaka, Bangladesh), respectively. BSO was extracted directly by cold pressing method (mentioned in the method section) from naturally obtained *Nigella sativa* seeds. Capmul MCM (C10) mono-/di-glycerides of capric acid was purchased from Abitec Corp. (Janesville, USA). Soybean oil (SBO; long chain triglycerides) was obtained from John L. Seaton and Co. Ltd. (North Humberside, UK). Maisine 35-1 (long chain mono-glycerides) was donated by Gattefosse SAS, (Saint Priest, France). Cremophor EL was purchased from BASF, (Ludwigshafen, Germany). Porcine pancreatin (8XUSP specifications) was purchased from Sigma Chemical Co. (St Louis, MO, USA). HPLC grade methanol, acetonitrile, formic acid, and phosphoric acid were obtained from BDH Chemicals Ltd., (Poole, UK). High purity deionized water was obtained through a Milli-Q Integral Water Purification System (Millipore, Bedford, MA). All other reagents were used as of analytical grade without further purification.

### 2.2. Experimental methods

#### 2.2.1. Development of SNEDDS and their components selection

A series of formulations was designed to be designated as nanoemulsifying systems and compared the performance with one another. A key objective was to use as few excipients as possible and keeping surfactant constant to ensure the performance of the materials as they were closely related. The representative SNEDDS formulations were selected from each type in considering the maximum drug loading capacity (SN and DN in combined dosage form).

The compositions of SNEDDS were prepared using different natural/semi synthetic long chain lipid oils, and hydrophilic surfactants which have good solubilizing capacity for the model drugs. All four SNEDDS formulations were studied using just four lipid components and one surfactant only; BSO, SBO, Maisene 35-1 (M35-1), Capmul MCM (CMCM, C_10_), and Cremophor EL (CrEL), respectively.

#### 2.2.2. BSO extraction and thymoquinone standardization

BSO (dark yellow in color), which was extracted from *Nigella sativa* seed, was collected from the Southwest part of Bangladesh in early summer of 2019. Approximately 500 g of the collected seeds were washed in fresh water, well dried under sunlight, and carried for oil extraction using cold pressed technique and preserved in a screw-capped amber colored glass bottle till further use. The main bioactive component of the BSO is thymoquinone which is chemically known as 2-isopropyl-5-methylbenzo-1, 4-quinone. The amount of thymoquinone was estimated by ultra-high-performance liquid chromatography (UHPLC) analysis mentioned in our previous studies (Kazi et al., 2020). The amount of thymoquinone in BSO was obtained as 20–50% which was in accordance with those reported in literature (Kazi et al., 2018; Alwadei et al., 2019).

#### 2.2.3. Emulsification efficiency assessment

The self-emulsification efficiency of SNEDDS was evaluated upon aqueous dilution with water. An amount of 100 mg of each formulation was added to 100 mL of milliQ water maintained at room temperature (22 °C). The diluted samples were given a gentle agitation by a standard stainless-steel spatula. ‘Spontaneity’ and ‘appearance’ were considered as assessment tools for visual observation of the prepared nanoemulsions. To predict the performance of the formulation *in vitro*, the following grading systems were used for evaluation: Grade A: Emulsion formation in less than 1 min with transparent appearance, Grade B: Emulsion formation in less than 1 min with bluish appearance and Grade C: Emulsion formation in less than 1 min with fine whitish appearance. The ratio of dilution 1:1000 was maintained for all the formulations.

#### 2.2.4. Self-emulsification time

To determine the self-emulsification time, each formulation (100 mg) was dropped into 10 mL of milliQ water maintained at room temperature (22 °C) in a glass beaker. The diluted samples were mixed gently using a star shaped magnetic stirrer (Metrohm^®^, Switzerland) rotating at constant speed (600 rpm). The time needed for an anhydrous formulation to form a homogeneous mixture upon dilution was recorded by manual stirring option from the software. The emulsification time was determined until the final appearance of the nanoemulsion was observed (Nasr et al., 2016).

#### 2.2.5. Droplet size distribution of liquid SNEDDS and polydispersity index

The droplet size distribution of diluted SNEDDS was measured by laser diffraction analysis using Zetasizer (NanoZS, Germany) particle sizing systems (Agarwal et al., 2009). Prior to measurement, the anhydrous SNEDDS were diluted with milliQ water at a ratio of 1:1000 *v/v* and well-mixed for 1 min. Samples were measured 10 times at room temperature after directly placing in the Zetasizer unit. All the experiments were carried out in triplicate to calculate the average volume particle size distribution and good correlation was found between the measurements. According to the published results earlier, the self-nanoemulsifying efficiency is strongly associated with the mean droplet size of the produced emulsion in aqueous systems (Atef & Belmonte, 2008). It is worth mentioning that the size range of the SNEDDS nanoparticle is able to retain its performance and clarity even after further aqueous dilution. Polydispersity determination of the SNEDDS formulation systems was calculated from a cumulants analysis of the dynamic light scattering (DLS)-measured intensity autocorrelation function. The polydispersity index (PDI) explains the width of the assumed Gaussian standard distribution where a single particle size mode and an exponential fit is applied to the autocorrelation function.

#### 2.2.6. Determination of zeta potential

The zeta potential was determined by measuring the diluted representative formulation using laser diffraction analysis by Zetasizer (Nano ZS, Germany) particle sizing systems. The formulations were diluted at a ratio of 1:1000 v/v (SNEDDS: milliQ water) as mentioned in droplet size analysis and mixed for 1 min before analysis. Emulsion droplet’s charges and their zeta potential values were recorded.

#### 2.2.7. Transmission electron microscopy (TEM)

The surface morphology and globule sizes of the representative SNEDDS formulation were observed using TEM (Jeol JEM1010, Tokyo, Japan). The sample was prepared upon 10 times dilution with milliQ water (SNEDDS: milliQ − 1:10 times). For analysis, a drop from the resulting nanoemulsion was placed on a film-coated copper grid to form a thin liquid film. These films were then negatively stained with 2% (w/v) phosphotungstic acid solution and then air-dried. The stained films were photographed by TEM.

#### 2.2.8. Equilibrium solubility studies

The equilibrium solubility of the two model drugs, SN and DN, was determined in the SNEDDS formulations using the simple shake flask method (Mohsin et al., 2016). The samples were prepared by adding an excess amount of SN and DN to the formulations, which was then thoroughly agitated with a vortex mixer to ensure adequate mixing. Three replicates were taken for each formulation. The samples for the solubility experiments were analyzed using in-house developed UHPLC method by dissolving each of the formulations with an appropriate solvent.

#### 2.2.9. Determination of the antioxidant activity

##### 2.2.9.1. Scavenging activity of DPPH radical

For antioxidant activity, DPPH (2, 2-diphenyl-1-picrylhydrazyl) free radical scavenging assay was employed. This method was carried out as previously described by Brand-Williams et al. (1995). The method quantifies free radical scavenging capacity of the investigated samples. DPPH is a molecule that contains a stable free radical. In the presence of an antioxidant which is able to donate an electron to DPPH, the change in the absorbance at 517 nm is followed spectrophotometrically (UV mini-1240, Shimadzu, Japan). Various concentrations of DPPH (10, 50, 100, 500, and 1000 μg/mL) were used. The assay mixture was made up of a total volume of 1 mL, containing 500 μL of the sample, 125 μL of the prepared DPPH, and 375 μL of methanol. As positive control, ascorbic acid was used. The samples were incubated for 30 min at 25 °C, after which the decrease in absorbance was measured at *λ* = 517 nm. Radical scavenging activity of the samples were calculated using the following equation below:

(1) % radical scavenging activity=(Abs control − Abs sample/Abs control) ×100

##### 2.2.9.2. β-carotene–linoleic acid assay

The antioxidant activity of the samples were evaluated using the *β*-carotene bleaching method described by Velioglu et al. (1998) with modifications. One milliliter (1 mL) of a 0.2 mg/mL solution of *β* -carotene in chloroform was added to flasks containing 0.2 mL of Tween 20 and 0.02 mL of linoleic acid. The chloroform was removed in a rotary evaporator at 40 °C. The resulting mixture was then diluted with 100 mL of distilled water and mixed for approximately 2 min to form an emulsion. Similarly, another mixture was prepared without *β*-carotene to use as a blank. A control containing 0.2 mL of 80% (v/v) methanol was also used instead of sample. Aliquot of the emulsion (5 mL) was added to 0.2 mL of the sample at 1 mg/mL. As a standard, Rutin (1 mg/mL) was used. The tubes were incubated in a water bath at 40 °C for 2 h. Absorbance was measured at 470 nm at 15 min intervals, using a UV–visible spectrophotometer (UV mini-1240, Shimadzu, Japan) and the antioxidant activity was calculated from the equation below:

(2) % of antioxidant activity= (Abs0 − Abst)/(Abs0° − Abst°) ×100

where, Abs0 and Abs0° represented the absorbance values measured at zero time of incubation for sample extract and control, respectively. Abst and Abst° are the absorbance values at *t* = 120 min for samples and control, respectively.

#### 2.2.10. Dynamic dispersion studies

SN and DN were dissolved in the representative SNEDDS at 80% loading based on their maximum equilibrium solubility determinations in the relevant anhydrous formulation. All the formulations investigated in the previous experiments were selected in the corresponding dynamic dispersion studies. The purpose was to examine if drug precipitation occurs during aqueous dispersion and to calculate the rate of precipitation. Accurately, 500 mg of formulation was dropped into 50 mL aqueous simulated intestinal media maintaining 1 in 100 dilution under fed (FeSSIF) and fated (FaSSIF) conditions and kept in a dry heat incubator at 37 °C for 24 h. Approximately, 1 mL of the dispersed sample from each container was withdrawn in an Eppendorf tube periodically during 24 h time, and centrifuged at 13,000 × *g*. A 100 µL aliquot of the clear supernatant was analyzed by the UHPLC to determine the drug amount (SN and DN) that remain solubilized in the sample. All of the experiments were performed in triplicate and the mean ± SD was calculated.

#### 2.2.11. *In vitro* digestion (lipolysis) experiments using pancreatic lipase

The lipolysis experiments were conducted by using 250 mg of F1 and F3 SNEDDS (SN and DN concentrations: 80% of the equilibrium solubility). The formulation was dispersed into 9 mL of simulated intestinal aqueous media under fed conditions [19, 21]. Phospholipids and bile salt were included in the lipolysis buffer at a molar ratio of 4:1, which is the actual ratio secreted in bile (20 and 5 mM). Prior to enzyme addition, emulsification of the representative SNEDDS was done for 5 min to form mixed micellar solutions in a thermostatic jacketed glass reaction vessel. The lipolysis reaction was started with the addition of 1 mL pancreatin solution (800 tributyrin units of pancreatic lipase) to reaction vessel. Lipolysis experiment was conducted at a constant pH of 6.8, using a pH-stat titration unit (Metrohm^®^, Switzerland) and continued for 30 min at 37 °C. Samples were periodically withdrawn at 0, 5, 10, 20, and 30 min during the titration reaction to examine the drug solubility in digests. A 0.5 M solution of 4-bromophenol boronic acid in methanol was used to stop further lipolysis of the withdrawn samples. Drug content from the aqueous phase were directly analyzed by UHPLC using suitable dilution with ACN.

#### 2.2.12. Anti-diabetic studies

##### 2.2.12.1. Animals

Male Swiss albino mice (20–25 g) roughly of same age group were procured from the Experimental Animal Care Center, College of Pharmacy, King Saud University, with the approval Clearance No. KSU-SE—19-105 (20th November 2019) by the Ethical committee. The animals used in this study were kept at 22 °C temperature by maintaining 55% humidity and light/dark (12/12 h) conditions. They were provided free access to Purina chow and drinking water ad libitum. The experimental rats were initially acclimatized for seven days prior to being used for the anti-diabetic studies.

##### 2.2.12.2. Induction of diabetes and experimental design

Diabetes was induced overnight in fasted mice by single intraperitoneal (IP) injection of 60 mg/kg of streptozotocin (STZ) that was freshly dissolved in in 0.1 M cold citrate buffer at pH 4.5. After 72 h of STZ administration, blood was collected from the animals and plasma glucose levels were examined. Animals that were confirmed diabetic by the elevated plasma glucose levels (>200 mg/dl) were used for the experiment. The animals were then randomly assigned into seven groups of three animals and received the following treatments: Group I: Normal control mice treated with Normal saline; Group II: Diabetic control mice treated with Normal saline; Group III: Diabetic mice treated with thymoquinone 5 mg/kg (IP); Group IV: Diabetic mice treated with DN 6 mg/kg (IP); Group V: Diabetic mice treated with SN 10 mg/kg (IP); Group VI: Diabetic mice treated with DN 6 mg/kg (IP) and SN 10 mg/kg (IP); Group VII: Diabetic mice treated with marketed product Dapazin^®^ 6 mg/kg (IP).

#### 2.2.13. *In vivo* oral bioavailability studies

##### 2.2.13.1. Animals

Male rats (Wister, 200–220 g) were obtained from Laboratory Animal Center of pharmacy college, KSU. The rats were randomly allocated into two groups, each group consisted of six rats: the representative SNEDDS [F1, BSO/CMCM/CrEL (15/35/50%w/w)] (group A) and marketed drug (group B). The animals were fasted for 12 h prior to the oral administration of SNEDDS of DN (6 mg/kg rat). The experimental procedures were carried out in accordance with the principles of National Institute of Health Guide for the Care and Use of Laboratory Animals (NIH Publications No. 80–23; 1996) as well as the animal facilities guidelines from the Ethical committee of Experimental Animal Care Center, College of Pharmacy, King Saud University (Clearance No. KSU-SE—19-105; 20th November 2019). The animals were housed in a temperature-controlled room with a 12-h light/dark cycle and were allowed access to food and water *ad libitum* during the study except that the chow was removed 12 h prior to treatment.

##### 2.2.13.2. Pharmacokinetic studies and experimental design

*In vivo* animal studies were performed using only DN in the dose and SN was not included due to the different pharmacokinetic features of the SN and DN. The bioavailability of the optimized SNEDDS in the study for DN was compared with marketed product. Approximately 0.5 mL of blood samples was collected from the retro-orbital plexus in a heparinized tube at different time intervals (at 0.0, 0.5, 1, 2, 4, 6, 12, 24, 36, and 72 h). Blood samples was then centrifuged at 5500 rpm for 15 min to separate the plasma was stored at −80 °C until analysis. The concentration of DN in plasma was determined by UHPLC as follow: A 775 μL of methanol was added to 225 μL aliquot of plasma. After vortex mixing for 1 min, the resultant was centrifuged at 5500 rpm for 10 min, and the organic layer was transferred to a clean tube and dried under a light stream of nitrogen at 45 °C. The residue was re-dissolved by 225 μL of mobile phase and 5 μL was injected by using HSS C18 (2.1 × 50 mm, 1.8 µm) analytical column. The nonlinear pharmacokinetic parameters such as *C*_max_, *T*_max_, and AUC_(0–∞)_ were determined.

##### 2.2.13.3. Pharmacokinetic assessment and UHPLC analysis of plasma samples

The pharmacokinetic assessment was done after oral administration on the basis of DN concentrations in the plasma samples of the rat. Extraction of the model drug, DN from the rat plasma was done by Liquid-Liquid Extraction procedure. Plasma samples collected were transferred into 1.5 mL Eppendorf tubes. A 1 μg/mL concentration of internal standard solution was constantly added to all the plasma samples along with methanol. The mixture was vortexed for 8–10 min and then centrifuged for 10 min at 4725 rpm. The clear supernatant containing the organic layer was transferred into clean centrifuge tube and dried under nitrogen gas at 45–50 °C. Dry residues obtained here were then reconstituted carefully in 225 μL of mobile phase. This was vortexed and transferred to an auto sampler vial to be analyzed in the UHPLC system.

Chromatographic separation of the DN from the plasma sample was simply performed on a Dionex^®^ UHPLC systems (Ultimate 3000, Thermo scientific, Bedford, MA, USA) with a photodiode array detector. The mobile phase was an isocratic mixer of methanol with (0.1% formic acid and 0.2% PhA) aqueous solution (28:72) % and ACN in a ratio of 82/18 v/v with flow rate of 0.4 mL/min. The total run time was 6 min with the sample injection volume of 2.0 µL for the analysis. This method for plasma analysis was validated based on the International Conference on Harmonization guidelines (Q2A I (R1), 2005). The linearity of the method was found to be suitable in the range of 100–10,000 ng/mL (*R*^2^ = 0.9999).

##### 2.2.13.4. Statistical analysis

Prism^®^ pad software was used to analyze the data. One-way analysis of variance followed by post hoc tests (LSD) was applied to compare the solubility and droplet size results. A value of *p* < .05 was considered significant throughout the study.

## 3. Results and discussion

The SNEDDS formulation in this study was developed using therapeutically active excipients (i.e. black seed oil). The BSO, containing thymoquinone as the active phytochemical (2-isopropyl-5-methylbenzo-1, 4-quinone) shows antioxidant and anti-inflammatory effects used to design representative SNEDDS in combined dosage form. It has been commonly used for more than 2000 years as a traditional remedy to treat a variety of health conditions (Goreja, 2003). Thymoquinone is the most hydrophobic bioactive molecule (549 to 740 µg/mL in aqueous solutions), thus if loaded externally into formulations, it could poses solubility challenges (Ali & Blunden, 2003; Salem, 2005; Salmani et al., 2014) for systemic absorption. The current studies minimizes this solubility challenges of thymoquinone as it is inert in the SNEDDS in addition to its synergistic effects in lowering glucose level inhibition and increasing antioxidant activities.

Selection of proper lipidic excipients remain the key challenge to successful formulation design, which also influences the bioavailability of the poorly soluble drugs along with the safety issues and costs. The formulation development process included the selection of the suitable excipients, such as bioactive/polar oils for SN and DN as combined dosage form in considering therapeutic value. In the current studies, it was clearly shown that the selection of a suitable bioactive excipient in SNEDDS could have the potential performance in enhancing the oral bioavailability and therapeutic effect of the model drugs, SN and DN, for diabetic patients.

Given the multiple pathophysiological lesions in type 2 diabetes, combination therapy remains a logical approach to control the disease management. The combined use of SN and DN in oral dose could be an effective treatment for type 2 diabetes mellitus, consistent with the complementary mechanisms of action by which these two agents improve glucose control (Jabbour et al., 2014). The expected achievements of the dosage form would be strongly correlated to the medical and health care area, particularly within the pharmacy and pharmaceutical sciences. These achievements could improve the quality of medication delivered to the diabetic patient. In addition, the improvement of loading capacity of two anti-diabetic drugs in single SNEDDS formulation, thus, is to reduce the dose frequency and pill burdens. In the current study, SNEDDS (contained thymoquinone from BSO) were successfully developed for the combined dosage form design, which included: (a) the role of lipid excipients in formulation design and development techniques, (b) maintaining both model drugs in solubilized state which provided the rationale for avoiding drug precipitation tendency from the lipid formulation systems through the gastro intestinal tract, and (c) the bioavailability of the SNEDDS using male Wister rats for the model drug.

### 3.1. Development of the SNEDDS formulation

**Figure 2.**
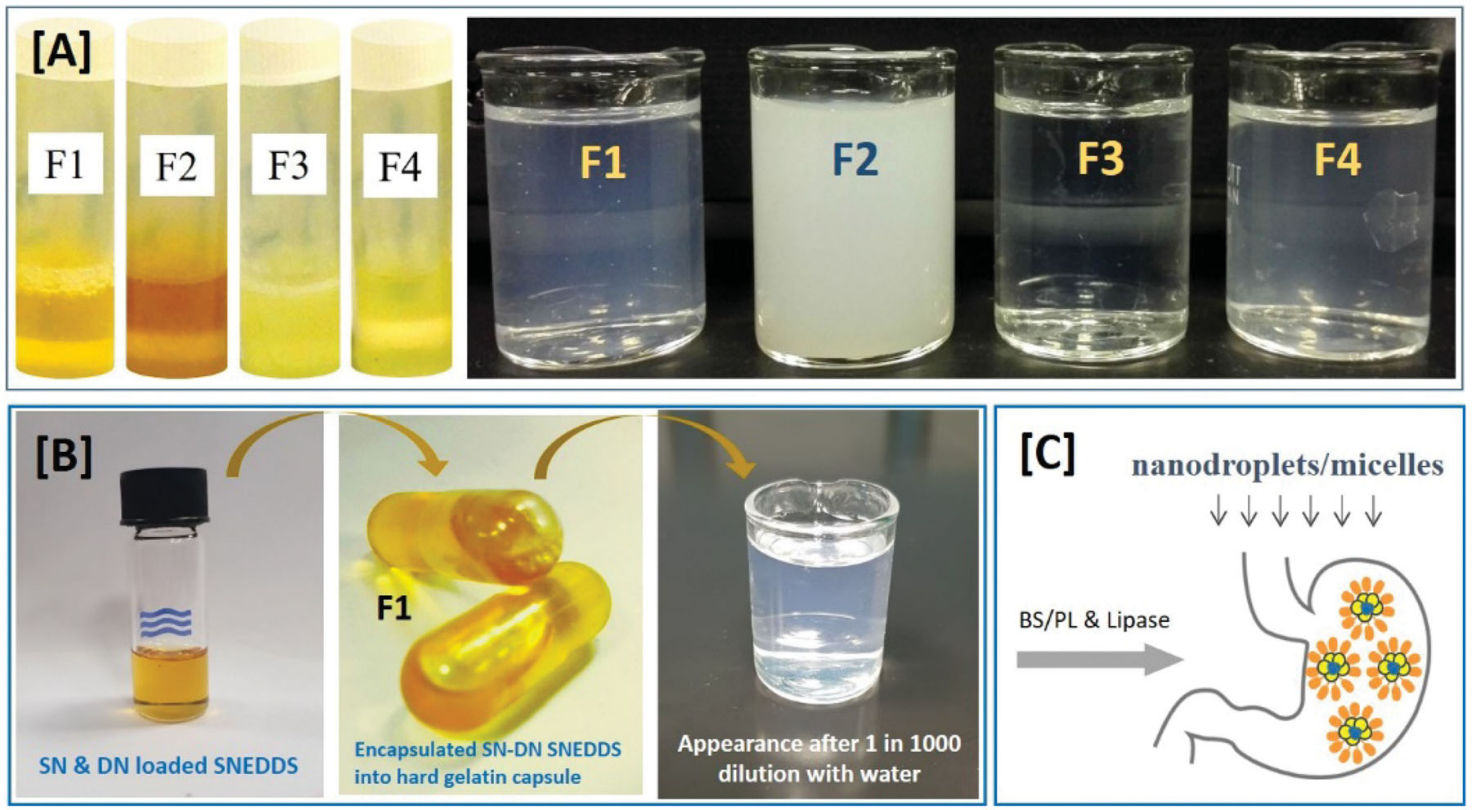
(A) Appearance of the SNEDDS preconcentrate (in glass tubes) and SNEDDS after aqueous dilution with water (in glass beaker) that were developed with black seed oil (BSO) and soybean oil (SBO) for the model drugs, sitagliptin and dapagliflozin, as combined dosage form. (B) The F1-SNEDDS combined dosage form filled into hard gelatin capsule and the appearance of the diluted SNEDDS (10 mg anhydrous formulation was diluted in 10 mL water [1 in 1000 w/v]) and (C) Formation of nanodroplets/micelles from F1-SNEDDS in presence of bile salt: phospholipid and pancreatic lipase during lipid digestion in the small intestine.

The excipients used to design SNEDDS in this research and the fraction used to blend together to yield various formulation systems are shown in (Table 1). Four formulations were developed using various concentrations of oil blends with one specific water soluble surfactant. Two long chain triglycerides oils such as BSO and SBO was blended with capmul medium chain mono-glycerides and maisene long chain mono-glycerides, respectively. The first two formulations (F1 and F2) contained 15% and 35% BSO and 35% and 15% capmul MCM with 50% nonionic surfactant cremophorEL. On the other hand, formulation F3 and F4 contained SBO 15% and 35% and maisene 35-1 35% and 15% with 50% cremophor EL. Nonionic surfactant cremophor EL used was constantly at 50% in all four formulations. The formulations were categorized in two different kinds as medium chain SNEDDS (F1 and F2) and long chain SNEDDS (F3 and F4) in the current set. The appearance of the anhydrous formulations and their dispersion upon aqueous dilution are shown in Figure 2. The anhydrous formulation of F1 and F2 produced yellowish colored texture due to the BSO. The aqueous dispersion of F1, F3, and F4 were shown transparent and or bluish appearance whereas F2 produced whitish appearance.

**Table 1.** The SNEDDS compositions were prepared using different concentrations of lipid oils and nonionic surfactant.

Formulation	% BSO	%SBO	%CMCM	% M35-1	% CrEL
F1	15	–	35	–	50
F2	35	–	15	–	50
F3	–	15	–	35	50
F4	–	35	–	15	50

BSO: black seed oil; SBO: soybean oil; CMCM: Capmul MCM; M35-1: Maisene 35-1; CrEL: Cremophor EL.

Surfactant was fixed at 50% of the total weight of the formulations.

Regarding the self-emulsifying efficiency, a visual assessment study is very common and a prerequisite, which could minimize the excess usage of chemicals due to the trial and errors (Kommuru et al., 2001). Therefore, miscibility of the oil/surfactant mixture in terms of hydrophilic lipophilic balance (HLB), homogeneity and appearance upon aqueous dilution were considered as visual assessment tools. The HLB values of the oils and surfactants can give a good indication of whether the two lipid excipients are miscible and should be used in the formulation or not. Within the scope of the current study, formulations that were homogeneous with clear/bluish appearance were considered to be efficient. Table 2 depicts the efficiency evaluation results. Although the excipients miscibility was promising in all four formulations, the appearance upon aqueous dilution were different along with emulsification times. Formulation F1, F3, and F4 were produced transparent appearance and considered to be the efficient SNEDDS based on their appearances. These formulations were tagged as grade A SNEDDS in the current studies.

**Table 2.** Performance parameter of the lipid-based self-nanoemulsifying formulations (SNEDDS) in terms of homogeneity/miscibility, appearance, and self-emulsification aptitude.

Formulation	Compositions (% w/w)	Emulsification time (s)	Appearance	Grade
F1	BSO/CMCM/CrEL [15/35/50]	10.45 ± 1.03	Transparent	A
F2	BSO/CMCM/CrEL [35/15/50]	7.45 ± 0.55	Whitish	B
F3	SBO/M35-1/CrEL [15/35/50]	23.50 ± 2.40	Transparent	A
F4	SBO/M35-1/CrEL [35/15/50]	16. 70 ± 1.50	Transparent	A

*Note*. All formulations were dispersed in less than a minute.

Visual observation which was a primary means of assessment to differentiate good and poor formulations, may be enough for an experienced formulator. By this observation, one can minimize a great deal of trial and errors in formulation design. Figure 2 showed that all four formulations were dispersed spontaneously with fine dispersing appearance when diluted with water at maximum dilution level.

### 3.2. Appearance of the SNEDDS formulation after aqueous dilution

Developed SNEDDS formulations were assessed for the physical appearance after dilution in aqueous media (water). The physical appearance of the SNEDDS suggests that the dispersion can be formed upon aqueous dilution in the gastro intestinal tract and thereby produce nanodroplets (Kazi et al., 2020). Initially dispersion was assessed visually after adding 1 g formulation to 100 mL water. Typically, the formulations produced transparent appearances spontaneously when diluted (Table 2). In the current formulation design study, the representative SNEDDS was only considered to be efficient if it was homogeneous and spontaneously dispersed (i.e. dispersion took less than 1 min, shown in Table 2). The efficiency of the formulation was visually evaluated using the following grading system (Nasr et al., 2016): Grade A: Spontaneously forming emulsion having a clear or bluish appearance (within 30 s, F1, F3, F4). Grade B: Spontaneously forming but slightly less clear emulsion, having a fine white appearance (F2). The results from the efficiency assessment showed that all the grade A SNEDDS were found to be promising in terms of the assessment criteria and were used for further developmental process (Figure 2).

### 3.3. Self-Emulsification time

The rate of emulsification is the prime determinant factor to estimate the efficiency of SNEDDS (Pouton, 2006). The recorded self-emulsification times for the drug loaded (SN and DN) formulation are presented in Table 2. From the data obtained, it was shown that all the tested formulation of SN-DN SNEDDS were self-emulsified within 7.45 to 23.50 s. This short period of self-emulsification as recorded in all the investigated formulation systems showed their potentiality for easy and rapid self-emulsification. The data from the self-emulsification assessment test also suggested that the rapid emulsification occurs only when the surface tension is reduced during the formulation dispersion and depends mainly upon the nature of the oil/surfactant and their proportion.

From the overall self-emulsification time, it was suggested that if the concentration of water-soluble excipients increased in the formulation, the spontaneity of emulsification process improved but self-emulsification time might decrease. In addition, the use of cremophor EL facilitated in reducing the interfacial tension and thus causing significant interfacial disruption and discharge of droplets into the bulk aqueous phase. These results were in agreement with the work done by Ali Nasr et al. in 2016, where SNEDDS were developed for Olmesartan medoxomil using surfactant cremophor RH40 and found that all representative SNEDDS formulations showed far less emulsification time in the range of 14 to 23 s (Nasr et al., 2016).

### 3.4. Droplet size analysis and PDI values of SNEDDS

As stated earlier, the self-emulsifying efficiency is strongly associated with the mean droplet size of the produced emulsion and on their stability. The droplet size analysis of the SNEDDS formulation showed lower particle sizes distribution upon aqueous dilution. The formulation F1 and F3 formed ultrafine dispersion with particle size of 66.57 ± 5.33 nm and 56.94 ± 3.87 nm and the systems were found to be a stable upon aqueous dilution, respectively (Table 3).

**Table 3.** Mean particle size (*Z* average), polydispersity index (PDI), and zeta potentials of the sitagliptin and dapagliflozin loaded liquid SNEDDS of different types.

Formulations %(w/w)	*Z* average (nm)	PDI	Zeta potential (mV)
BSO/CMCM/CrEL [15/35/50]	66.57 ± 5.33	0.140	–16.10 ± 4.77
BSO/CMCM/CrEL [35/15/50]	112.00 ± 10.35	0.380	–13.25 ± 2.54
SBO/M35-1/CrEL [15/35/50]	56.94 ± 3.87	0.046	–22.56 ± 5.12
SBO/M35-1/CrEL [35/15/50]	160.44 ± 12.10	0.570	–15.32 ± 4.48

PDI – polydispersity index is a determinant of the heterogeneity of particle sizes in a mixture, the value is the mean of three measurements.

SNEDDS contain several components which may affect the droplet size in GI fluids upon dispersion. Therefore, the droplet size analysis was essential and could mimic the dilution behavior *in vivo.* The results from the droplet size analysis suggested that more the water-soluble materials in the formulation, lower the droplet size substantially upon aqueous dispersion. Hence, the droplet sizes of F1 and F3 were less than F2 and F4 due to high contents of water-soluble excipients in the formulations. In addition, the SNEDDS were widely monodispersed in the aqueous media with lower polydispersity values of less than 0.2.

The PDI is dimensionless and scaled in a way that values smaller than 0.05 are considered to be highly monodispersed. Values greater than 0.7 indicate that the sample has a very broad size distribution and is probably yield large droplet sizes in the DLS technique. The small values of PDI shown by F1 and F3 SNEDDS formulation (0.140 and 0.046) indicated a homogenous droplet population and narrow globule size distribution as shown in Table 3.

### 3.5. The zeta potential measurement

The potential stability of the SNEDDS in aqueous system is determined by the zeta potential values. Zeta potential measurement is one of the quick tests to shorten stability studies of the candidate formulations, hence reducing the experimental time and testing cost as well as improving shelf-life.

Zeta potential is the charge that develops at the interface between a solid surface and its liquid medium. If the dispersed particles of aqueous media have high positive or negative zeta potential values, then they will repel each other to create dispersion stability. On the other hand, substantially low zeta potential values of the dispersant cannot force to prevent the particles coming together and therefore become unstable. Particles with zeta potential values of –16.10 ± 4.77 mV and –22.56 ± 5.12 mV for F1 and F3, respectively are considered stable. The zeta potential values of other SNEDDS were in the range of – (13.00–15.00) mV as shown in Table 3. It was reported that the concentration of cremophor EL could have steric stabilization capacity of the SNEDDS system by forming a coat around their surface (Mohsin & Pouton, 2012). Usually, the nonionic surfactants do not contribute any charges to the nanoemulsion particles rather stabilizing the systems and this indicated that negatively charged particles contribute to the stability of formed nanoemulsion. The measured value for zeta potential is more than 15 mV which indicates good electrical properties for the preparer SNEDDS.

The overall results suggest that a potential zeta value of ≈–16.10 mV has been able to form a physically stable nanoparticle through the mechanism of forming a protective layer around the droplet from the pull of bonding together the dispersing medium to avoid the unification of SNEDDS droplet.

### 3.6. Observation of physical stability

Appearance of the dispersant (F1 and F3) showing the stability of the formulation after aqueous dispersion at initial dispersion time and after 3 months. F1 showed slight opaque color after 3 months without leading any sedimentation precipitation of SN and DN in anhydrous formulations. The anhydrous F1, F3 SNEDDS were kept for 3 months at room temperature for stability studies and, then, characterized in terms of their visual clarity, droplet size changes, and zeta potential values.

**Figure 3.**
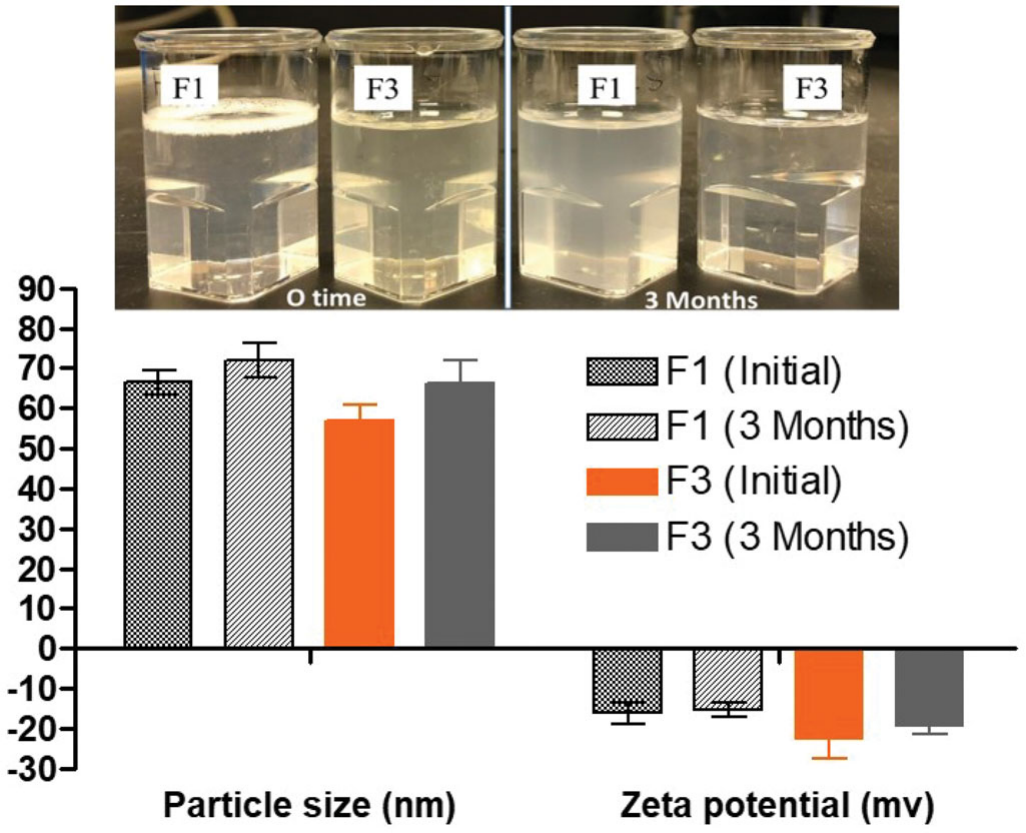
The appearance and the stability data (particle size and zeta potential values) of representative SNEDDS upon aqueous dispersion at initial and after 3 months of storage.

Physical stability was carried out to determine the maximum storage duration which can lead to the separation of emulsion (cracking) phases (Figure 3). The test results showed no significant droplet size changes and zeta potential values, which suggested good physical stability in both anhydrous and dispersed forms.

### 3.7. Transmission electron microscopy

**Figure 4.**
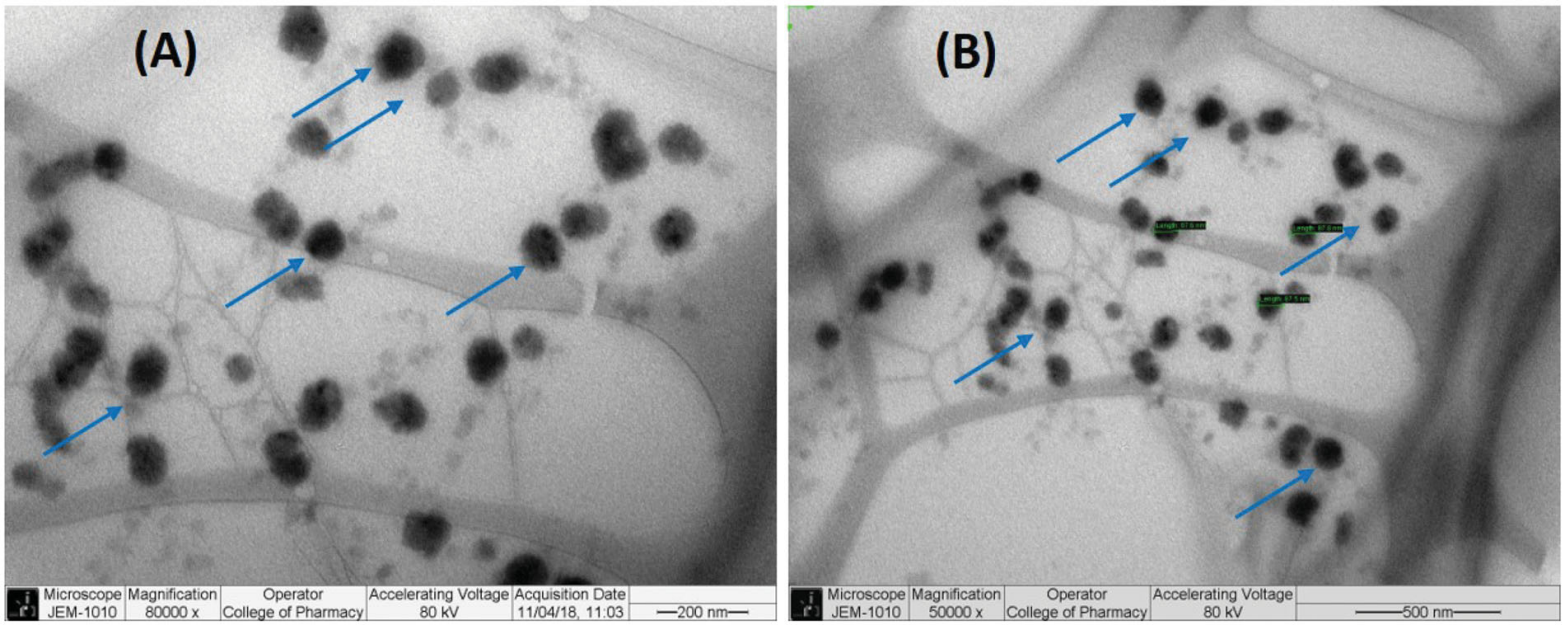
TEM micrographs of SN and DN loaded representative SNEDDS (BSO/CMCM/CrEL [15/35/50%w/w]). The arrows indicate the oil droplets.

TEM images of SN and DN loaded SNEDDS formulation subsequent to post dilution with deionized water are shown in Figure 4, which interpreted the surface morphology and globule size. From the Figure 4(A), it was apparent that globules of the representative formulation were well dispersed without aggregation. TEM data (Figure 4(B)) suggest that SN-DN loaded F1-SNEDDS showed spherical and homogeneous droplets and were in the nanometer size range (50–100 nm), which was also evident by the particle size distribution data obtained using Malvern Zetasizer (Table 3). The uniformed droplet size of the monodispersed self-nanoemulsifying formulation in the current study was well-correlated by the images obtained from TEM analysis.

### 3.8. Equilibrium solubility of SN and DN in anhydrous formulation

The therapeutic efficiency of DN is particularly limited by its poor water solubility and low bioavailability after oral delivery. In this study, all four SNEDDS were studied to improve the solubility of SN and DN in combined dosage form. During the initial screening process, a suitable SNEDDS formulation of SN and DN, was selected in terms of the following important considerations: (1) compatibility of the excipients within the formulation compositions, (2) good solubility of SN and DN, and (3) the efficient droplet size and stability after forming nanoemulsion (Zhang et al., 2008). Therefore, the selection of oils and surfactant as well as the mixing ratio of oil to surfactant played an important role in SNEDDS formulation development.

The solubility of SN and DN in anhydrous SNEDDS were shown in Table 4. The components used in the SNEDDS formulation should solubilize the maximum amount of drug and should possess a high efficiency in self-emulsification.

**Table 4.** Equilibrium solubility (mg/g) of sitagliptin and dapagliflozin in various anhydrous, and diluted self-nanoemulsifying lipid formulations.

Formulation	Solubility (mg/g) at 0%, 90%, 99%, and 100% water					
	Dapagliflozin			Sitagliptin		
	0%	90%	99%	0%	90%	99%
F1	55.07 ± 2.95	8.49 ± 0.99	0.90 ± 0.03	11.14 ± 0.90	39.93 ± 1.32	6.11 ± 0.05
F2	44.38 ± 4.32	5.25 ± 1.01	0.71 ± 0.02	5.83 ± 0.55	34.48 ± 0.78	5.01 ± 0.09
F3	28.53 ± 3.21	3.99 ± 1.20	0.38 ± 0.09	8.88 ± 0.96	38.56 ± 1.22	7.32 ± 0.10
F4	25.84 ± 1.23	3.07 ± 0.85	0.275	6.93 ± 0.81	29.87 ± 0.90	5.67 ± 0.07
Water (100%)	1.48 ± 0.14 mg/g			3.62 ± 0.11 mg/g		

Amongst all the anhydrous formulations, F1-SNEDDS, BSO: CMCM (3:7) with CrEL at ratio [50:50% w/w] exhibited the highest SN and DN solubility at 11.14 mg/g and 55.07 mg/g (*p* < .05), respectively with transparent appearance upon aqueous dilution. Formulations (F2), which contained the same oil components as F1 but opposite mixing ratios [7:3] with 50% surfactant cremophor EL, respectively showed the comparably lower solubility of SN and DN (5.83 mg and 44.38 mg) in anhydrous formulations. These formulations were also found to be transparent and bluish in appearance. On the other hand, the SNEDDS containing long chain lipids such as SBO and maisene 35-1 at ratio 3:7 and 7:3 with 50% cremophor EL as surfactant have lower equilibrium solubility. The solubility studies suggest that both SN and DN are highly soluble in the presence of polar lipids (high percent of mono-glycerides) in the formulations.

Taken together, result from the solubility study showed that the highest drug solubility and better aqueous dispersibility was exhibited by F1-SNEDDS among all formulations, and this F1-SNEDDS system has been chosen for the *in vivo* animal studies and anti-diabetic studies. It was also confirmed that both SN and DN prefer the SNEDDS which contained more medium chain polar glycerides and water-soluble surfactant.

The main purpose of the solubility measurement was to increase the loading capacity of two anti-diabetic drugs in single SNEDDS formulation, thus, to reduce the dose frequency and improve the oral bioavailability. Table 4 shows the SN and DN solubility performance with 1 g SNEDDS formulation having capacity to dissolve 11.14 mg and 55.07 mg SN and DN, respectively. The higher solubility of SN and DN in SNEDDS formulation suggests that the mixture of glycerides (blend of mono-glycerides and di-glycerides- BSO: CMCM), along with hydrophilic surfactant, cremophor EL (polyoxyl 35-hydrogenated castor oil).

### 3.9. Antioxidant activity

The antioxidant activity assay showed that the representative SNEDDS (F1: BSO/CMCM/CrEL [15/35/50] %w/w) possess moderate antioxidant activity in both methods (DPPH scavenging and *β*-carotene bleaching assay) when they are compared with the standards (Ascorbic acid and Rutin). The result from the study shown in Table 5 suggested that thymoquinone is the main constituent of the BSO (*N. sativa* seeds), which was reported to have antioxidant activity. It has retained its antioxidant activity when formulated with model drugs, SN and DN, in the current study.

**Table 5.** Antioxidant properties of the representative SNEDDS (F1: BSO/CMCM/CrEL [15/35/50] %w/w), drug free SNEDDS and pure drugs.

Sample	Radical scavenging activity in (%)					Total antioxidant activity in (%)
	10 µg	50 µg	100 µg	500 µg	1000 µg	1000 µg
F1-SN&DN SNEDDS	19.2 ± 2.4	35.2 ± 2.9	41.4 ± 2.3	69.3 ± 2.7	74.3 ± 1.5	76.4 ± 2.1
F1 Drug free SNEDDS	NT				17.6 ± 2.4	18.7 ± 2.4
Pure SN&DN	0.05	0.08	0.33	0.69	1.30	1.61
Ascorbic acid	33.7 ± 3.5	74.1 ± 1.3	85.6 ± 3.2	88.7 ± 2.7	91.7 ± 3.4	NT
Rutin	NT	NT	NT	NT	NT	93.1 ± 1.2

Total antioxidant activity in (%): β-carotene bleaching assay, NT not tested. In the columns, mean ± SD with different letters notification are significant at (*p* < .05; *n* = 3).

### 3.10. Mass in solution expected from equilibrium solubility study

**Figure 5.**
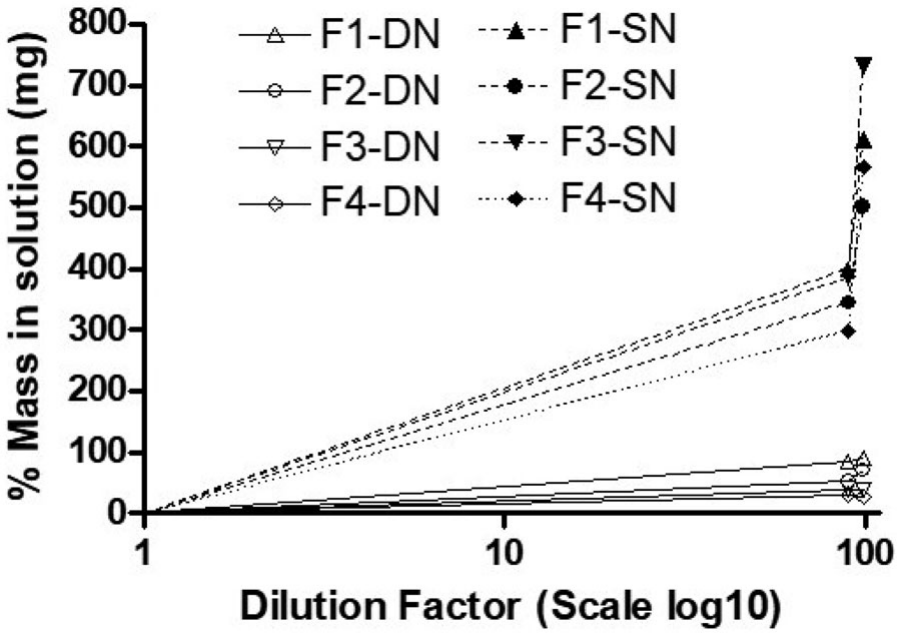
Theoretical mass (mg) of drug which can be dissolved at thermodynamic equilibrium after dilution of the four (F1–F4) SNEDDS formulations with 90% and 99% water. All the SNEDDS are able to load significant mass of SN in the diluted formulations (*p* < .05 compared to mass of DN).

In our previous studies, it was found that the determination of the equilibrium solubility of drug in the diluted formulation can be a useful tool of anticipating real-time drug precipitation. Hence, this experiment was conducted to investigate drug precipitation or on the other hand drug solubilization capacity after dilution of lipid formulations. The results in Figure 5 showed that all the diluted SNEDDS (F1, F2, F3, and F4) have significantly higher SN solubility compared to DN. However, among all the SNEDDS, F1-SN and F3-SN (99% diluted formulation) have high aqueous solubility (*p* < .05). The overall results suggested that SN can be loaded more that 400% of the aqueous solubility in the anhydrous SNEDDS, which was confirmed by its high drug loading capacity in aqueous media (Figure 5).

### 3.11. Dynamic dispersion profiles of SN-DN

The release performance of the SNEDDS formulation and absence of drug precipitation upon dispersion is the desired goal for pharmaceutical applications. Dynamic dispersion tests for the developed SNEDDS *in vitro* was used to examine the ability of SNEDDS vehicle when dispersed into intestinal media and therefore helped us to follow the roles in development and selection of appropriate SNEDDS formulations for further *in vivo* studies for the combined dose. The extensively improved solubilization capacity of the SNEDDS formulation over the desired drug concentration could help avoid *in vivo* drug precipitation.

**Figure 6.**
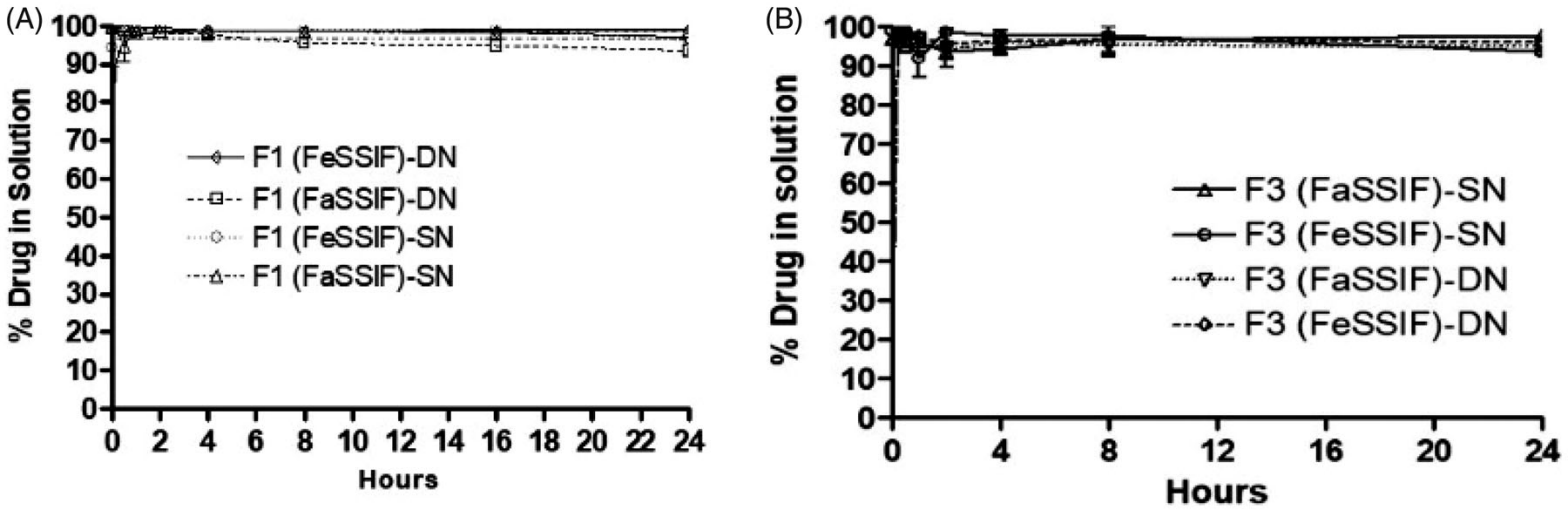
(A) % Drug release of sitagliptin (SN) and dapagliflozin (DN) from F1-SNEDDS combined dosage form (8.91 mg and 44.06 mg, 80% of the equilibrium solubility of SN and DN, respectively) in simulated intestinal media (FaSSIF and FeSSIF). (B) % Drug release from F3-SNEDDS combined dosage form (7.10 mg and 22.82 mg, 80% of the equilibrium solubility of SN and DN, respectively) in FaSSIF and FeSSIF. Arithmetic means (*n* = 3) are shown, standard deviations are within the symbols.

*In vitro* dispersion tests appropriately predicted whether precipitation is likely to occur prior to absorption. In the current assessment, the SN and DN loaded SNEDDS were tested in the Fasted (FaSSIF) and Fed State Simulated Intestinal Fluid (FeSSIF) and the drug concentration was monitored until 24 h time. The results showed that the F1-SNEDDS containing 80% SN and DN (8.91 mg and 44.06 mg) maintained more than 99% drug in solution within 4 h (Figure 6(A)) without leading any noticeable precipitation. Similarly, the F3-SNEDDS (SN and DN loaded 7.10 and 22.82 mg), maintained more than 95% drugs in solution until 24 h time (Figure 6(B)).

The dispersion results for F1 and F3 confirmed that polar mixed glycerides of the proposed SNEDDS formulations can retain high percent drugs in solution for more than 24 h time in the fasted and fed state intestinal media (FaSSIF and FeSSIF).

The lack of available data to describe the relationship between formulation and performance *in vivo* has been a disadvantage to the formulation of lipid-based delivery systems. SNEDDS that disperse quickly giving an ultrafine nanoemulsion/microemulsion are transparent with a transparent/bluish appearance. However, a more important characteristic of the nanoemulsion is the ability of the formulation to maintain the drug in solution through transit within the gastrointestinal tract. To achieve this, the drug must be kept in solubilized state during dispersion, which typically occurs in the stomach, as well as during stomach digestion and subsequently digestion in the small intestine. As such, we focused on the dispersion phase in the stomach and examine how the excipients used in the SNEDDS formulation influences the rate of precipitation.

### 3.12. Investigation of *in vitro* lipolysis (digestion) of SN-DN

**Figure 7.**
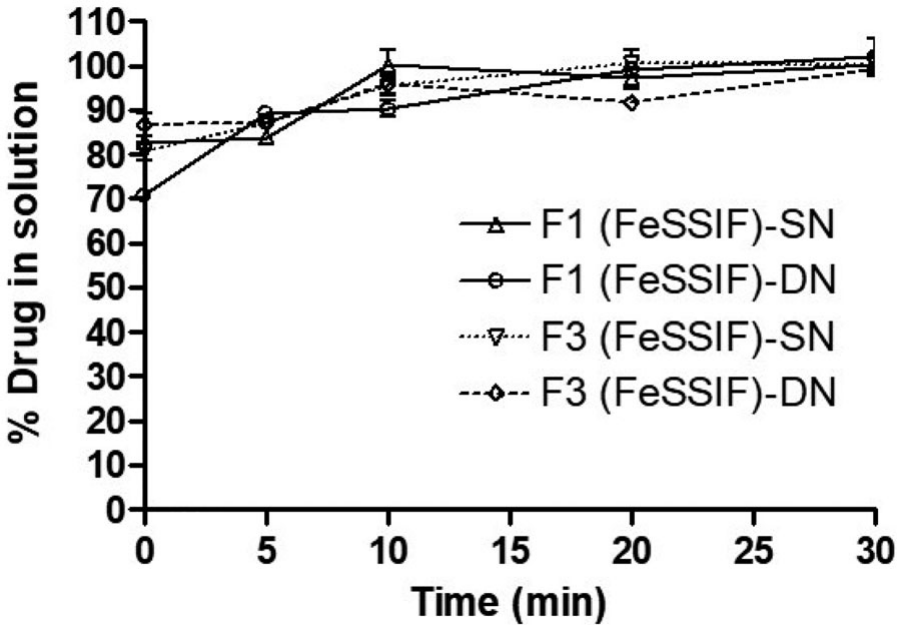
% Drug in solution in presence of pancreatic lipase from the representative SNEDDS formulations (F1 and F3) which contain SN and DN as combined dosage form in FeSSIF solution during the *in vitro* digestion experiment at various time intervals within the 30 min reaction period.

The purpose of the *in vitro* lipolysis study was to see if any precipitation occurred during the lipolysis test period of 30 min. The results in Figure 7 showed that the concentrations of SN and DN were found approximately 85% at 5 min, which suggested incomplete release of the drugs in the digests. However, the 100% drug release was achieved after completion of the lipolysis reaction for 30 min in presence of pancreatic lipase/co-lipase. There was not any precipitation recorded for both SN and DN which was supported by the above dynamic dispersion results in the current studies. However, it has been reported by Thomas et al. that *in vitro* lipolysis data does not predict adequately the *in vivo* performance of a self-nanoemulsifying lipid-based drug delivery systems (SNEDDS; Thomas et al., 2014). Furthermore, SNEDDS formulation are very flexible to produce diverse dosage forms such as liquid palatable solution, and conversion into solid dosage forms (self-emulsifying tablets, multiparticulate beads, and pellets etc.). Therefore, liquid SNEDDS have been remained as extensively demanding and effective alternatives to conventional emulsion or tablets.

### 3.13. Anti-diabetic studies

The results from the anti-diabetic studies showed that after 2 h of dose intake, the blood glucose level was significantly reduced with the combined dose of SN and DN using F1-SNEDDS (63.97%) compared to SN alone (54.54%), DN alone (39.50%; *p* < .05), and marketed drug (45.05%; Table 6). Table 6 showed the levels of glucose inhibition in serum in each group. It was reported that BSO (thymoquinone) containing optimized F1-SNEDDS resulted in reduced fasting glucose, decreased insulin resistance, increased beta-cell function, and reduced glycosylated hemoglobin (HbA1c) in human subjects (Abdelrazek et al., 2018). Compared with SN or DN monotherapy and marketed product, DN plus SN loaded SNEDDS showed better anti-diabetic efficacy in the diabetic mice. The overall studies suggest that the combination therapy of SN, DN with thymoquinone (an additional therapeutic compound included from BSO) had considerable effect in lowering glucose, as such it may also be associated with improved beta-cell function and enhanced insulin clearance in addition to their established underlying mechanisms of action.

**Table 6.** Anti-diabetic activity of sitagliptin and dapagliflozin in diabetic mice.

Treatments	Dose (mg/kg)	Glucose level mg/dl			
		0 h	2 h	Level decrease (mg/dl)	% Inhibition
Control (normal mice)	N. saline	120.66 ± 2.18	117.00 ± 3.60	3.66	3.03↓
Control (Diabetic mice-STZ)	N. saline	384.00 ± 13.65	382.66 ± 12.03	1.33	0.34↓
Diabetic mice (STZ) + Blank SNEDDS	60 mg + 6 mg	360.66 ± 6.64	353.33 ± 11.02	7.33	2.03↓
Diabetic mice (STZ) + DN (pure)	60 mg + 6 mg	362.00 ± 4.93	219.00 ± 7.57	143	39.50↓
Diabetic mice (STZ) + SN (Pure)	60 mg + 10 mg	381.33 ± 11.25	173.33 ± 7.53***	208	54.54↓
Diabetic mice (STZ) + SN-DN-SNEDDS	60 mg + 6 mg + 10 mg	379.66 ± 7.26	136.66 ± 3.93***	242.66	63.97↓
Diabetic mice (STZ) + Marketed (Tab)	60 mg + 6 mg	361.00 ± 6.42	198.33 ± 8.11***	162.66	45.06↓

Data are presented as (Mean ± SEM, *n* = 3). ***p < .001, followed by Students *t*-test.

**Figure 8.**
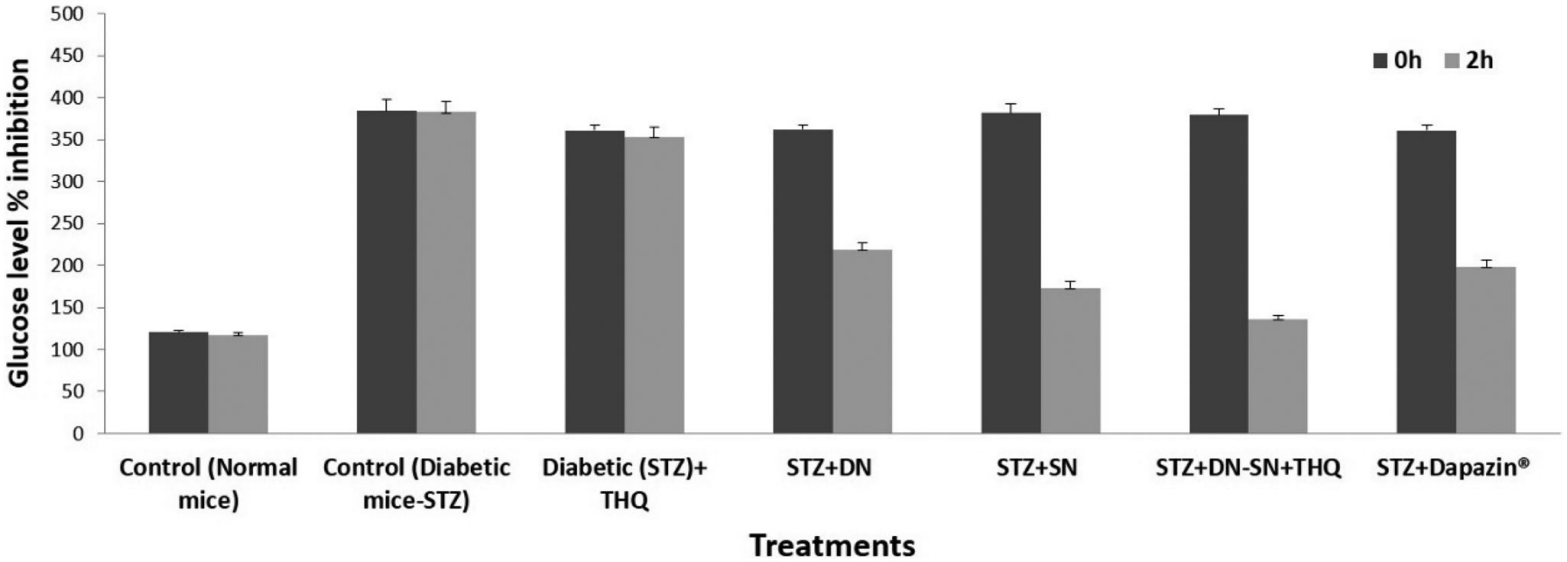
% Glucose level inhibition of sitagliptin and dapagliflozin from normal control, diabetic (STZ) control, diabetic (STZ) + THQ, diabetic (STZ) + DN, diabetic (STZ) + SN, diabetic (STZ) + DN + SN (F1-SNEDDS-BSO/CMCM/CrEL [15/35/50] %w/w combined dose), diabetic (STZ) + marketed drug. STZ: streptozotocin; SN: sitagliptin; DN: dapagliflozin; THQ: thymoquinone. Data are presented as (Mean ± SEM, *n* = 3).

It was suggested that the first model drug SN was active in stimulating the secretion of insulin, and the second drug DN prevented the absorption of filtered glucose from the kidneys and that excreted through urine. Therefore, the glucose was not reabsorbed into the blood causing high blood glucose levels. On the other hand, the presence of black seed extract (thymoquinone) in the combined representative F1-SNEDDS provided extra therapeutic properties in reducing blood glucose level (Figure 8) and in reducing insulin resistance.

### 3.14. Oral bioavailability studies

**Figure 9.**
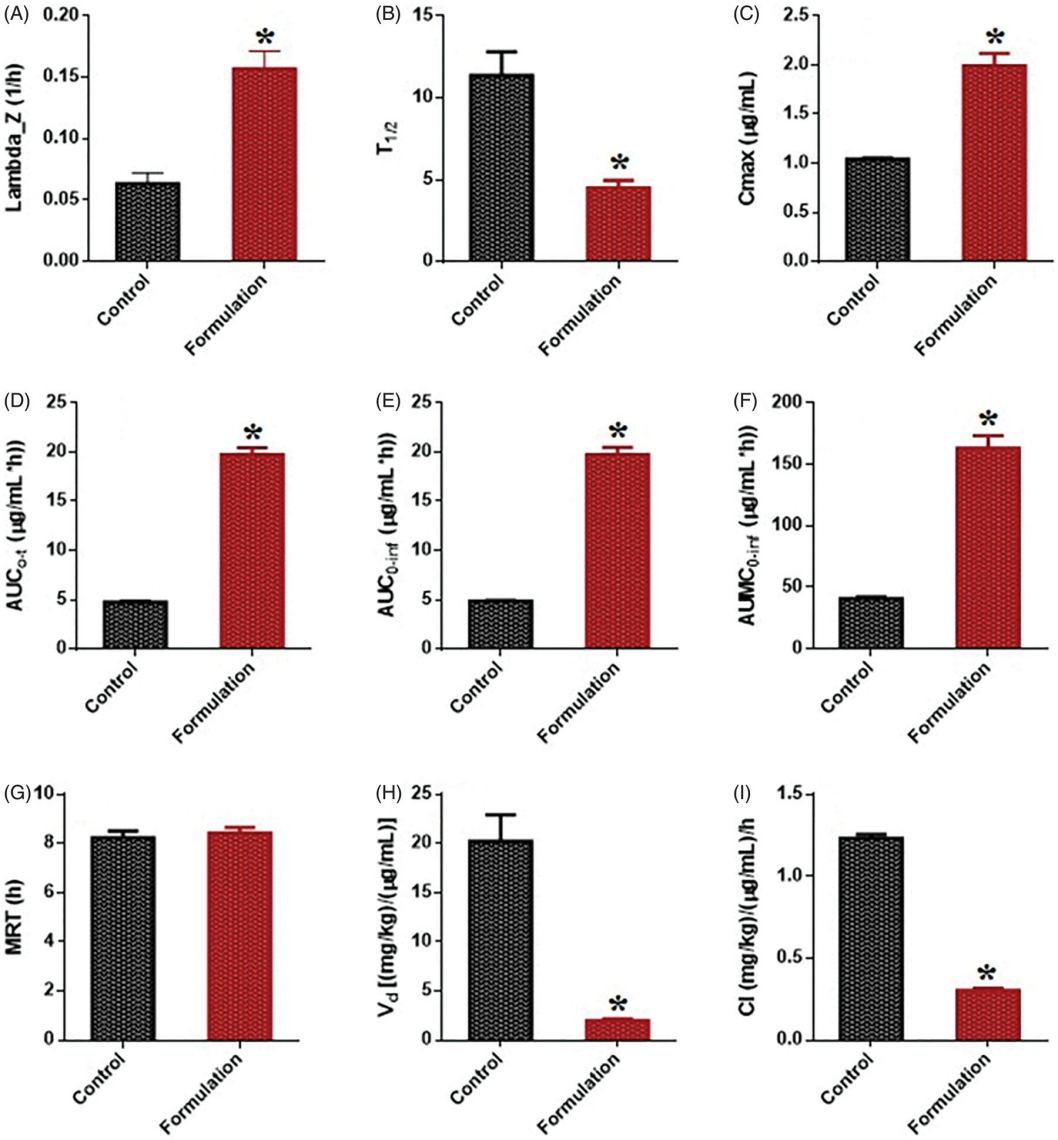
Pharmacokinetic profiles of dapagliflozin (DN) after a single oral administration of formulation (F1-SNEDDS: BSO/CMCM/CrEL [15/35/50%w/w]) and control (marketed drug) to male Wister rats at a dose equivalent to 6 mg/kg dapagliflozin (Mean ± SEM, *n* = 6). * denotes the significance as *p* < .05. *C*_max_: peak of maximum concentration; AUC_0→t_: area under the concentration time profile curve up to time 24 h; AUC_0→∞_: area under the concentration time profile curve extrapolated to infinity; *V*_d_: volume of distribution at steady state; *T*_1/2_: half-life; MRT: mean residence time; CL: oral clearance.

The *in vivo* pharmacokinetic behavior of the representative F1-SNEDDS formulation was studied to quantify DN at the present investigation in rat plasma after oral administration. DN is reported in this study to be well absorbed after oral administration, with peak plasma levels attained within 4 h. Figure 9 showed that the *C*_max_ of the DN from the oral administration of marketed product was found to be 1.04 ± 0.02 µg mL^−1^, whereas the *C*_max_ of DN from the oral administration of our developed F1-SNEDDS formulation was 1.99 ± 0.21 µg mL^−1^. The *C*_max_ value of DN from the SNEDDS formulation was significantly increased from 1.04 ± 0.02 to 1.99 ± 0.21 21 µg mL^−1^ (*p* < .05).

The AUC of DN was also significantly increased in the SNEDDS treated group as compared to only marketed product treated group from 6.46 ± 0.17 to 17.94 ± 1.25 (µg mL^−1^). The *in vivo* study exhibited a twofold increase in the oral bioavailability of DN from our F1-SNEDDS compared with the marketed product (tablet). These results suggest the potential use of the proposed SNEDDS for improving oral bioavailability of anti-diabetic drug DN, which could be a great choice for combined dose with SN to improve the treatment of Type 2 diabetes.

Findings reported from the antidiabetic studies here support that DN and SN in a combined dosage form showed better efficacy and improved outcomes in reducing blood glucose level. We however investigated only DN for its *in vivo* pharmacokinetic studies. The reason was to correlate the enhancement of solubility and percentage of solubilized drug with the enhancement of its bioavailability, which was essential for the product performance. This would construct a proper *in vitro* and *in vivo* correlation for the drug of similar nature (Kazi et al., 2018). The bioavailability enhancement of DN may be due to the increased solubility and faster uptake of the nanoemulsion resulting from SNEDDS formulation by enterocytes at the absorption site. These findings from DN were agreed in accordance with several previous reports and expected to be the same for SN (Zhang et al., 2014; Kazi et al., 2019).

## 4. Conclusions

The representative SNEDDS formulated in the current studies provide collective advantages such as superior self-emulsification efficiency with improved physical stability and antioxidant activity, high drug loading capacity, high glucose inhibition level, and elevated DN bioavailability. The most appropriate excipient’s concentrations that able to produce representative SNEDDS system were BSO (contains phytochemical thymoquinone) 15%, Capmul MCM 35%, and Cremophor EL 50%, respectively. The overall results suggest that the proposed SN-DN loaded F1-SNEDDS, which improve glucose control with an extra benefit from thymoquinone could be delivered as the potential combined dosage form encapsulated into hard and or soft gelatin capsules for the effective treatment of type 2 diabetes mellitus.
